# Person-centered care in Norwegian nursing homes and its relation to organizational factors and staff characteristics: a cross-sectional survey

**DOI:** 10.1017/S1041610217002708

**Published:** 2017-12-04

**Authors:** Irene Røen, Øyvind Kirkevold, Ingelin Testad, Geir Selbæk, Knut Engedal, Sverre Bergh

**Affiliations:** 1 Centre for Old Age Psychiatric Research, Innlandet Hospital Trust, Ottestad, Norway; 2 Norwegian National Advisory Unit on Ageing and Health, Vestfold Hospital Trust, Tønsberg, Norway; 3 Department of Health Science in Gjøvik, Norwegian University of Science and Technology (NTNU), Gjøvik, Norway; 4 Centre for Age-Related Medicine – SESAM, Stavanger University Hospital, Stavanger, Norway; 5 Department of Old Age Psychiatry, Institute of Psychiatry, Psychology & Neuroscience, King's College London, London, UK; 6 Medical School, St Luke's Campus, University of Exeter, Exeter, UK; 7 Faculty of Medicine, University of Oslo, Oslo, Norway

**Keywords:** cross-sectional, nursing home, person-centered care, organizational factors, job satisfaction, physical environment

## Abstract

**Background:**

Person-centered care (PCC) is regarded as good quality care for persons with dementia. This study aimed to explore and understand the association between PCC and organizational, staff and unit characteristics in nursing homes (NHs).

**Methods:**

Staff from 175 NH units in Norway (n = 1,161) completed a survey, including measures of PCC and questions about staff characteristics and work-related psychosocial factors. In addition, data about organizational and structural factors and assessment of the physical environment in the units were obtained. The distribution of these factors in regular units (RUs) and special care units (SCUs) is described, and the differences between the two types of units are analyzed. Furthermore, multilevel linear regression analyses explored the extent to which variables were associated with PCC.

**Results:**

Higher levels of PCC were associated with a greater job satisfaction, three years or more of health-related education, a lower level of quantitative demands and role conflict, a higher level of perception of mastery, empowering leadership, innovative climate and perception of group work, in addition to the type of unit and the physical environment in the NH unit designed for people with dementia. SCU and staff job satisfaction explained most of the variation in PCC.

**Conclusion:**

This study shows an association between PCC and organizational, staff and unit characteristics in NH. These findings indicate that providing PCC in NH care is closely linked to how the staff experiences their job situation in addition to both organizational and structural factors and the physical environment. Attention needs to be given to such factors when planning NH care.

## Introduction

Dementia is a syndrome caused by a variety of brain disorders, which leads to cognitive decline and decreased function in the activities of daily living. The syndrome is usually chronic and progressive in nature. As dementia increases in severity, the need for institutionalization increases, and more than 80% of Norwegian nursing home (NH) patients have dementia (Selbaek *et al.*, 2007).

Furthermore, dementia is a condition compounded of the personality of the person with dementia, and his or her physical health, life story, neurologic impairment, and social psychology, all preserving the person's personhood (Kitwood, 1997). According to Kitwood (1977), the person with dementia and their psychological needs is the focus of the care and treatment; person-centered care (PCC) (Kitwood, 1997) rather than the person's disease (Edvardsson and Innes, 2010). PCC is widely accepted as good-quality care for persons with dementia in NH and is a guiding principle in care services (Brooker, 2004; Li and Porock, 2014; Manthorpe and Samsi, 2016). There is an increase in the literature evaluating psychosocial interventions and PCC (Li and Porock, 2014; Testad *et al.*, 2014) showing significant benefits on decreasing behavioral symptoms (Chenoweth *et al.*, 2009), psychotropic medication use (Fossey *et al.*, 2006), increase in mood (Brooker *et al.*, 2011), and health-related quality of life (Ballard *et al.*, 2015) in persons with dementia in long-term care. Theories for implementing PCC have been developed over the years, such as the VIPS framework by Brooker (2004). This framework constitutes four major elements; *V* stands for valuing people with dementia and those who care for them; *I* for treating people as individuals; *P* for looking at the world from the perspective of the person with dementia; and *S* for a positive social environment in which the person living with dementia can experience relative well-being (Brooker, 2004).

In PCC, the relationship between NH staff and the person with dementia is essential (Wilberforce *et al.*, 2016), and consequently, the staff's attitude and work methods are important (Anderson *et al.*, 2016). Several factors have in previous studies been associated with PCC, such as job satisfaction and capacity to provide individualized care (van den Pol-Grevelink *et al.*, 2012; Brownie and Nancarrow, 2013), gender, beliefs about personhood in dementia, burnout, collaboration in care, the physical environment, the social environment of care (Hunter *et al.*, 2015), and the psychosocial factors at work (Testad *et al.*, 2010). A recent review article concluded that the physical environment in care settings is important for improving the patients’ quality of life and quality of care practices (Chaudhury *et al.*, 2017). Essential aspects of quality of life and care include the influence of unit size, the spatial layout, its homelike character, sensory stimulation, and specific areas for dining, bathing, and outdoor activities, all of which emphasize the relationship between the therapeutic physical environment, organizational factors, and care practices (Chaudhury *et al.*, 2017). Although the published work on PCC is quite substantial, the number of included respondents is low and the need for larger studies is warranted. Furthermore, insight on how organizational structures can stimulate or hinder person centeredness in staff and whether levels of person centeredness correlate with individual staff variables, such as education, clinical experience, job satisfaction, and experience of organizational variables, such as type of ward (regular unit (RU) or special care unit (SCU)), unit size, leadership, staff-to-patient ratios, and physical environment, is needed.

Thus, we designed this study to explore and understand the association between PCC, assessed with the Person-centered Care Assessment Tool (P-CAT), and organizational, NH staff and unit characteristics.

## Methods

### Study design and sample

This is a cross-sectional study, with a convenience sample of 175 NH units from 45 NHs in 29 municipalities in four Norwegian counties. An NH unit participating in the study was defined as a group of patients living together with a common living area and having their own care staff during the daytime. NH staff, which the head nurse defined as those familiar to the care provided and the structural and organizational conditions in the unit, were considered eligible for the study.

### Data collection

The data were collected during the period from October 2013 to December 2014. Three case report files (CRF), including both a standardized questionnaire and questions developed for this study by the research group, were constructed; one to the NH manager, one to the head nurse of the unit, and one to the NH staff. The members of the research group all have wide experience in both clinical work and research projects in NHs. The questions developed by the research group for the study were based on factors identified in the literature referring to organizational and structural factors, such as culture, leadership, management, staff education, staffing levels, and physical environment.

## Measures

### Person-centered care

Several tools have been developed to assess PCC (de Silva, 2014; Wilberforce *et al.*, 2016), but the P-CAT (Edvardsson *et al.*, 2010) is the only tool designed for self-assessing PCC by staff in long-term care facilities, which has been tested beyond the initial development stages (Wilberforce *et al.*, 2016). The Norwegian version of the P-CAT has satisfactory psychometric properties for the use in a nursing home-care setting (Rokstad *et al.*, 2012) and was chosen in this study. The P-CAT consists of 13 items expressed as statements about the content of care, the environment, and the organization, formulated to measure staff perceptions of the practice in the unit where they work. The participants indicate on a five-point Likert scale ranging from 1 (disagree completely) to 5 (agree completely) how they perceive the care in the unit. The total score ranges from 13 to 65, where higher scores indicate a higher level of PCC.

### NH staff factors

NH staff data were obtained through questionnaires. The questionnaire contained demographic information about the participants, such as age, gender, Norwegian as a first language, number of years of health-related and relevant continuing education, experience in the current job, and percentage of full-time position.

Work-related psychosocial factors were assessed with the General Nordic Questionnaire for Psychosocial and Social Factors at Work (QPS-Nordic), covering essential social and psychological factors at work (Dallner *et al.*, 2000). Of the 129 items in QPS-Nordic, 11 are background items, 38 are single items, and 80 are distributed in 13 scales. In this study, 32 items distributed in the following 10 scales were included: quantitative demands, decision demands, learning demands, perception of mastery, empowering mastership, fair leadership, role clarity, role conflict, innovative climate, and perception of group work. Respondents indicated how relevant each statement was for their situation on a five-point Likert scale from 1 (very seldom or never) to 5 (very often or always). Each scale consists of 3 or 4 items, giving a subscale score of 3–15 or 4–20 (see textbox 1).

**Textbox 1.** Organizational and psychosocial factors*

Subscales	QPS-Nordic items
Quantitative demands	– Is your work load irregular so that the work piles up?
	– Do you have to work overtime?
	– Is it necessary to work at a rapid pace?
	– Do you have too much to do?
Decision demands	– Does your work require quick decisions?
	– Does your work require maximum attention?
	– Does your work require complex decisions?
Learning demands	– Are your work tasks too difficult for you?
	– Do you perform work tasks for which you need more training?
	– Does your job require that you acquire new knowledge and new skills?
Perception of mastery	– Are you content with the quality of the work you do?
	– Are you content with the amount of work that you get done?
	– Are you content with your ability to solve problems at work?
	– Are you content with your ability to maintain a good relationship with your coworkers at work?
Empowering leadership	– Does your immediate superior encourage you to participate in important decisions?
	– Does your immediate superior encourage you to speak up, when you have different opinions?
	– Does your immediate superior help you develop your skills?
Fair leadership	– Does your immediate superior distribute the work fairly and impartially?
	– Does your immediate superior treat the workers fairly and equally?
	– Is the relationship between you and your immediate superior a source of stress to you?
Role clarity	– Have clear, planned goals and objectives been defined for your job?
	– Do you know what your responsibilities are?
	– Do you know exactly what is expected of you at work?
Role conflict	– Do you have to do things that you feel should be done differently?
	– Are you given assignments without adequate resources to complete them?
	– Do you receive incompatible requests from two or more people?
Innovative climate	– Do workers take initiatives at your workplace?
	– Are workers encouraged to think of ways to do things better at your workplace?
	– Is there sufficient communication in your department?
Perception of group work	– Do you appreciate belonging to this group or team?
	– Is your group or team work flexible?
	– Is your group or team successful at problem solving?

*Thirty-two QPS-Nordic items, distributed in 10 scales were used in the study. Each scale consists of 3 or 4 items, giving a subscale score of 3–15 or 4–20.

A single question about general job satisfaction was added: “How will you describe your general experience of your job satisfaction?” The alternatives were “very bad – bad – unsure – quite good – good – excellent.”

To ensure staff anonymity, the head nurse did not have access to the staffs’ answers in the questionnaires, with NH staff returning the questionnaire in a stamped envelope directly to the researchers.

### NH unit characteristics

The following data about organizational and structural factors in the NH unit were obtained through a questionnaire distributed to the head nurse of the 175 units: type of unit (SCU or RU); the unit size (number of patients); the daytime staff/patient ratio (the number of NH staff working per patient during the daytime); the number of units per head nurse; and the number of hours the nursing home physician was working per patient per week in the nursing home/unit. To categorize NH units as either RU or SCU, we used the definition of SCU from the Therapeutic Environment Screening Survey for Nursing Home (TESS-NH) (Sloane *et al.*, 2002): an SCU must be physically separated from the rest of the facility by closed doors or it is free-standing, and the unit must self-designate the unit as a specialized dementia care unit. In addition, the unit must meet two of the following three criteria: (1) the unit serves a population in which 75% or more of the residents have a diagnosis of Alzheimer's disease or related dementias; (2) unit programming and activities are dementia specific; or (3) the staff is trained in dementia care. NH units not fulfilling this SCU definition were defined as RUs, including regular somatic units, short-time units, and rehabilitation units.

### Physical environment

To assess the physical environment of the unit, we used the Special Care Unit Environmental Quality Scale (SCUEQS), which is a summary scale embedded in the Therapeutic Environment Screening Survey for Nursing Homes (TESS-NH) (Sloane *et al.*, 2002). TESS-NH was translated into Norwegian and back-translated according to the procedures described by Acquadro *et al.*(1996). Three translators, two medical doctors, and one registered nurse translated the American version of TESS-NH into Norwegian. These translations were aggregated into one Norwegian version, and a faculty research group agreed on a preliminary version. This version was translated back into English by Allegro Language Services. The English back-translated version was sent to Sloane, who developed the original TESS-NH, to get her comments. The final Norwegian version of TESS-NH was agreed upon after a revision based on Sloane's responses and a discussion in the research group. The Norwegian version is not tested for psychometric properties. The TESS-NH contains 84 discrete items and one global rating and was developed to describe the ability of physical environments in NHs to address therapeutic goals for persons with dementia. The SCUEQS consists of 18 of the TESS-NH items and measures maintenance, cleanliness, safety, lighting, physical appearance/home likeness, orientation/cueing, and noise (Sloane *et al.*, 2002). Scores range from 0 to 41, where higher scores indicate a better physical environment.

## Statistical analyses

IBM SPSS Statistics for Windows, version 23.0 (Armonk, NY: IBM Corp.) was used to perform descriptive statistics of P-CAT scores, QPS-Nordic and quality indicators, and staff and unit characteristics.

Of the 1,161 respondents, 77 had missing data on at least one P-CAT item and imputation was performed on cases with fewer than 50% missing values (6 at most). Four respondents had missing data on more than six P-CAT items, and data were not imputed. The empirical distribution for each item in the scale was generated. A random number was drawn from that distribution and used to replace the missing value. The process was repeated until all missing values were imputed. This algorithm mimics the bootstrap described by Efron and Tibshirani (1994).

As data were on two levels (unit and staff level), MLwiN version 2.36 (Centre for Multilevel Modeling, University of Bristol) was used to check for a clustering effect (Intra-Class Correlation (ICC)) of the units. After a clear cluster effect was found, three multilevel linear regression models were built using P-CAT sum scores as the dependent variable. Independent variables were added to the model in blocks: NH-staff characteristics (model 1), QPS-Nordic (model 2), and variables collected at unit level (level 2, model 3). The multilevel analysis generates two different values for variance σ^2^_en_ for between groups and σ^2^_un_ for within groups, and with this the proportion of the ICC explained by the models (R^2^_2_) and of the portion of variance within groups(R^2^_1_) were calculated at each step.

## Results

### Staff characteristics and work-related psychosocial factors

Characteristics of nursing staff are presented in Table 1. The total staff response rate was 67.5%. The mean staff response rate within the units was 70.7% (SD 20.3%), indicating that the response rates were lower in the large units. Nearly all the NH staffs were female (96.6%), 56.4% were between 40 and 59 years old, and 91.9% had Norwegian as their first language. Most of the staff (60.8%) had a position of 75% of full time or more, 29.9% had 3 years or more of health-related education, and 27.4% had received relevant continuing education. Regarding work experience, the largest group was those who had worked 5–15 years at the NH unit (49.7 %). Finally, 69.2% rated their job satisfaction as good or excellent.

**Table 1.** Characteristics of nursing staff n = 1,161

	n/%
Female Gender n =1,098	1,061/96.6
Norwegian as first language n =1,113	1,023/91.9
Age n = 1,136	
<20	8/0.7
20–29	155/13.6
30–39	187/16.5
40–49	295/26.0
50–59	346/30.5
60–67	133/11.7
>67	12/1.1
Years of health-related education n = 1,157	
≥3	346/29.9
<3	811/70.1
Relevant continuing education n = 1,161	
Yes	318/27.4
No	843/72.6
Experience in current job n = 1,123	
<1 year	84/7.5
1–4.99 years	262/23.3
5–14.99 years	457/40.7
15 years and more	320/28.5
Staff working at least 75% of full-time n = 1,151	700/60.8
Job satisfaction n = 1,151	
Very poor	5/0.4
Poor	18/1.6
Unsure	38/3.3
Quite good	293/25.5
Good	550/47.8
Excellent	247/21.5
QPS-Nordic^1^ subscales^2^	n/mean (SD^3^)
QPS-N, quantitative demands (4 items)	1,149/11.23 (2.84)
QPS-N, decision demands (3 items)	1,149/10.10 (1.99)
QPS-N, learning demands (3 items)	1,149/7.21 (1.76)
QPS-N, perception of mastery (4 items)	1,149/16.24 (1.93)
QPS-N, empowering leadership (3 items)	1,150/8.74 (2.96)
QPS-N, fair leadership (3 items)	1,151/11.87 (2.66)
QPS-N, role clarity (3 items)	1,151/13.00 (1.95)
QPS-N, role conflict (3 items)	1,151/7.38 (2.13)
QPS-N, innovative climate (3 items)	1,153/11.57 (2.10)
QPS-N, perception of group work (3 items)	1,145/12.12 (2.01)

1 QPS-Nordic = the General Nordic Questionnaire for Psychosocial and Social Factors at Work.

2 QPS-Nordic subscales each consist of 3 or 4 items, giving a subscale score of 3–15 or 4–20.

3 SD = standard deviation.

### Unit characteristics and person-centered care assessment

All leaders of the units except one returned the questionnaire, giving a response rate of 99.5%. Of the 175 units, 62 (35.4%) were SCUs. Table 2 presents differences between the RUs and the SCUs in the number of beds, the physical environment, staffing ratio, and the number of units the head nurse was leader of. All P-CAT scores except for one item – the environment feels chaotic – were higher in SCUs than in RUs. Four SCUEQS items – cleanliness of social spaces and halls, visual stimulation opportunities, and resident appearance – had higher scores in RU than in SCU, for all other SCUEQS items SCU had higher scores. Three items – floor surface in halls, light intensity in resident's rooms, and public areas homelike – in addition to the total sum score were significantly higher in SCU, indicating that SCU have a more dementia-friendly environment.

**Table 2.** Unit^1^ characteristics and P-CAT score in regular and special care units

variables	all n/mean (sd^2^)	regular unit n/mean (sd)	special care unit n/mean (sd)	p-value
Number of beds in unit	175/10.86 (5.68)	113/12.16 (6.42)	62/8.48 (2.72)	<0.001^5^
Staff/patient ratio	174/0.32 (0.09)	113/0.30 (0.06)	61/0.35 (0.13)	0.007^5^
Head nurse for number of units	174/2.81 (1.23)	113/2.60 (0.90)	61/3.20 (1.60)	0.009^5^
Physician; minutes per resident per week	174/26.09 (18.52)	113/25.03 (16.47)	61/28.05 (21.84)	0.348^5^
SCUEQS^3^				
Maintenance				
7a Maintenance of social spaces	175/1.35 (0.67)	113/1.32 (0.67)	62/1.40 (0.66)	0.405^6^
7b Maintenance of halls	175/1.39 (0.68)	113/1.34 (0.68)	62/1.50 (0.67)	0.093^6^
7c Maintenance of resident rooms	175/1.34 (0.68)	113/1.31 (0.72)	62/1.40 (0.61)	0.526^6^
7d Maintenance of resident bathrooms	175/1.31 (0.72)	113/1.23 (0.74)	62/1.45 (0.65)	0.063^6^
Cleanliness				
8a Cleanliness of social spaces	175/1.32 (0.64)	113/1.33 (0.63)	62/1.31 (0.64)	0.842^6^
8b Cleanliness of halls	175/1.37 (0.61)	113/1.37 (0.62)	62/1.35 (0.60)	0.826^6^
9a Bodily excretion odor in public areas	175/1.84 (0.43)	113/1.84 (0.43)	62/1.84 (0.41)	0.845^6^
9b Bodily excretion odor in residents rooms	175/1.82 (0.40)	113/1.81 (0.39)	62/1.82 (0.43)	0.722^6^
Safety				
10b Floor surface in halls	175/1.08 (0.83)	113/0.98 (0.88)	62/1.26 (0.74)	0.045^6^
Lightning				
12b Light intensity in activity areas	173/1.30 (0.68)	111/1.24 (0.95)	62/1.29 (0.64)	0.736^6^
12c Light intensity in residents rooms	175/0.98 (0.67)	113/0.90 (0.67)	62/1.13 (0.64)	0.031^6^
Visual/tactile stimulation				
25b Visual stimulation opportunities	175/1.86 (0.83)	113/1.90 (0.79)	62/1.79 (0.91)	0.458^6^
Noise				
31d Load speaker/intercom noise	172/1.91 (0.40)	112/1.88 (0.46)	62/1.97 (0.26)	0.176^6^
Familiarity/homelikeness				
19 Public areas homelike	173/1.42 (1.02)	111/1.24 (0.95)	62/1.73 (1.09)	0.005^6^
20 Kitchen in the unit	175/0.64 (0.89)	113/0.60 (0.88)	62/0.71 (0.89)	0.373^6^
21 Pictures/mementos in residents room	175/2.81 (0.58)	113/2.78 (0.64)	62/2.87 (0.46)	0.375^6^
23 Resident appearance	175/1.97 (0.18)	113/1.98 (0.13)	62/1.94 (0.25)	0.105^6^
Orientation				
28 c+d Current or old picture of resident	175/0.06 (0.24)	113/0.06 (0.24)	62/0.06 (0.25)	0.947^6^
SCUEQS total sum	175/25.72 (4.79)	113/25.15 (4.61)	62/26.76 (4.50)	0.033^5^
P-CAT^4^ total sum	1,157/46.16 (7.46)	763/44.82 (7.43)	394/48.74 (6.83)	<0.001^5^

1 A unit is defined as a group of residents living together with a common living area and having their own care staff during daytime.

2 SD = Standard deviation.

3 SCUEQS = Special Care Unit Environmental Quality Scale.

4 P-CAT = Person-Centered Care Assessment Tool.

5 *t*-test.

6 Mann–Whitney *U* test.

### Variables associated with P-CAT score

Multilevel linear regression analyses were conducted to analyze the associations between staff variables; QPS-N variables, unit variables, and the P-CAT score (Table 3).

**Table 3.** Multilevel linear regression with person-centered care assessment tool (P-CAT) sum as dependent variable

				model 1		model 2		model 3	
				n = 1,026		n = 1,002		n = 996	
		univariate		data from		data from		data from	
		analysis		175 units		175 units		174 units	
*Staff variables*	n	coefficient	p-value	coefficient	p-value	coefficient	p-value	coefficient	p-value
Age	1,132	−0.206	0.175	0.063	0.704	0.079	0.601	0.046	0.761
Gender	1,094	0.507	0.642	0.821	0.423	0.882	0.332	0.797	0.377
Job satisfaction	1,148	3.235	<0.001	3.275	<0.001	1.451	<0.001	1.453	<0.001
3 years or more health-related education	1,153	0.234	0.571	0.840	0.050	1.333	0.001	1.289	0.001
≥ 75% of full time position	1,135	0.183	0.641	−0.506	0.212	−0.543	0.135	−0.477	0.189
Experience in profession^1^	1,119	−0.285	0.197	−0.340	0.148	−0.243	0.254	−0.212	0.317
Advanced education	1,157	0.653	0.132	0.992	0.021	0.674	0.082	0.59	0.125
*QPS-Nordic*^2^									
QPS-N. Quantitative demands	1,145	−0.916	<0.001			−0.644	<0.001	−0.601	<0.001
QPS-N. Decision demands	1,145	−0.085	0.391			0.222	0.021	0.173	0.069
QPS-N. Learning demands	1,145	−0.509	<0.001			0.023	0.840	0.015	0.894
QPS-N. Perception of mastery	1,145	1.210	<0.001			0.260	0.015	0.260	0.015
QPS-N. Empowering leadership	1,146	0.759	<0.001			0.341	<0.001	0.343	<0.001
QPS-N. Fair leadership	1,147	0.879	<0.001			0.061	0.462	0.075	0.360
QPS-N. Role clarity	1,147	0.931	<0.001			0.009	0.934	0.034	0.753
QPS-N. Role conflict	1,147	−0.969	<0.001			−0.286	0.003	−0.281	0.003
QPS-N. Innovative climate	1,150	1.379	<0.001			0.515	<0.001	0.523	<0.001
QPS-N. Perception of group work	1,142	1.338	<0.001			0.453	<0.001	0.443	<0.001
*Unit variables*	*n*								
Type of unit^3^	175	3.825	<0.001					1.773	0.002
Number of beds	175	−0.168	0.007					−0.026	0.555
SCUEQS^4^ sum	175	0.181	0.020					0.142	0.005
Staff/patient ratio	174	10.607	0.007					4.361	0.109
Head nurse/number of units	174	0.438	0.158					0.303	0.172
Physician (minutes per patient)	174	−0.008	0.703					−0.006	0.668
ICC^5^ = 0.341									
R₁² (Within units)				0.166		0.348		0.346	
R₂² (Between units)				0.298		0.591		0.722	

1 Experience in profession (current job) in groups in years; 0 = ≤1, 1 = >1–4.99, 2 = 5–14.99, 3 = ≥15.

2 QPS-Nordic = The General Nordic Questionnaire for Psychosocial and Social Factors at Work.

3 Type of unit; 0 = Regular Unit (RU), 1 = Special Care Unit (SCU).

4 SCUEQS = Special Care Unit Environmental Quality Scale.

5 ICC = Intra-Class Correlation Coefficient.

High job satisfaction was associated with a higher P-CAT score in the univariate analysis, as well as all the three models in the multivariate analysis. Having three years or more health-related education was not associated with a higher P-CAT score in the univariate analysis or in model 1 of the multivariate analysis, but was associated with a higher P-CAT score in models 2 and 3 of the multivariate analysis, compared to having lower education.

In the univariate analysis, all the QPS-N subscales, except decision demands, were associated with the P-CAT score. Adjusted for staff variables and the other QPS-N subscale scores (model 2), decision demands, perception of mastery, empowering leadership, innovative climate, and perception of group work were positively associated with the P-CAT score, while quantitative demands and role conflict were negatively associated with the P-CAT score. This pattern was sustained in model 3 where unit variables were added to the model, except that decision demands were no longer significant.

In the univariate analysis, type of unit, number of beds, SCUEQS sum, and staff at daytime/patient were all associated with the P-CAT score. Adjusted for all the other variables (model 3), SCUs were associated with a higher P-CAT score compared to RUs, and a higher SCUEQS sum was associated with a higher P-CAT score.

## Discussion

The main finding of this study was that high job satisfaction in care staff and care organized in small, specialized units were both strongly associated with a high level of PCC. More specifically, we found that staff with three years or more of health-related education, a lower level of quantitative demands and role conflict, a higher level of perception of mastery, empowering leadership, innovative climate, and perception of group work, in addition to a physical environment in the NH unit designed for people with dementia, were all associated with higher levels of PCC. To our knowledge, this is the first study exploring the complex association between PCC, organizational, NH staff and unit characteristics in Norwegian NH.

The strongest association with high levels of PCC was a high score on job satisfaction, even when adjusted for all the QPS-Nordic subscales. This finding is supported by a previous review article from 2013 (Brownie and Nancarrow, 2013), which reported that facility-specific PCC interventions were found to impact the nurses’ sense of job satisfaction, a Dutch study which analyzed the association between PCC and job satisfaction, and concluded that PCC may contribute to higher job satisfaction (van den Pol-Grevelink *et al.*, 2012), and, finally, a Swedish study, which also found that higher levels of staff job satisfaction were associated with higher levels of PCC (Sjogren *et al.*, 2015). Together, these findings should be taken into account when planning care for residents in NH, even though we were not able to show if the staff's job satisfaction leads to higher degree of PCC or if it is the other way around.

In the univariate analysis, both the size of the units (number of beds) and the daytime staff/patient ratio were associated with higher levels of PCC, but these associations vanished when adjusted for other factors. However, SCUs were associated with higher levels of PCC, and as the average sizes of the SCUs were smaller and SCUs had on average a higher daytime staff/patient ratio (Table 2), both the size of the unit and the staff/patient ratio were important elements explaining the association between the type of ward and PCC.

Previous studies comparing SCUs and RUs have looked at patient outcomes, such as behavior, functioning in activities of daily living, cognitive function, and quality of life, to our knowledge, no other studies have specifically investigated the effect of SCU on PCC. A review from 2013 shows that patient characteristics only to a minor extent are different in SCU compared to RU (Kok *et al.*, 2013). Other studies have looked at quality indicators such as the use of restraints and the prescription of antipsychotics (Kirkevold and Engedal, 2008), and provision of case conferences (Palm *et al.*, 2016). These studies show that the literature is not consistent according to quality indicators. A review from 2009 evaluating the effect of SCUs concluded that it is probably more important to implement best practices than to provide a specialized care environment (Lai *et al.*, 2009). The definitions of an SCU vary across countries and across studies, and nursing homes implement different features in the units (Palm *et al.*, 2014), thus there are challenges in comparing studies of SCUs. In the present study, the average size of an SCU was 8.5 beds, while in a Canadian study the average size of the SCUs was 18.9 beds (Morgan *et al.*, 2004).

A Norwegian study concluded that SCUs had fewer quality deficiencies, probably due to the smaller units and a higher staff ratio (Kirkevold and Engedal, 2008). Together with the strong association between SCU and a high level of PCC, we argue that smaller, more homelike units, with a higher staff ratio, which are dedicated to persons with dementia, are a better option for persons with dementia than RUs.

Additionally, this study shows that not only the size of the unit, but also education and job satisfaction in care staff, leadership, and environment, impact the quality of care for this vulnerable group of persons. Care staff with three years or more of health-related education had a stronger association with a higher level of PCC in this study, similar to what was reported by Sjøgren *et al.* (2015), who found that a higher proportion of staff with continuing education in dementia care at the ward were associated with higher levels of PCC. With an aging population and the warranted decrease of care staff, this finding shows that not only do we need more staff, we need more staff with high education to care for our elderly.

Quantitative demands and role conflicts were both negatively associated with PCC in the present study. Similarly, Sjøgren *et al.*, who explored the relationship between PCC and the staffs work environment and job-related well-being, reported that NH units where staff felt supported by their leaders had higher levels of PCC (Sjogren *et al.*, 2015). Willemse *et al.* also found that leader support is associated with the nursing staff's person centeredness (Willemse *et al.*, 2015). To create a work environment where the leaders support the NH staff, it is important to provide PCC in NH.

The QPS-Nordic subscale perception of mastery was positively associated with PCC. This association is again supported by Sjøgren's study, which found that a lower level of stress of conscience among staff was associated with higher levels of PCC (Sjogren *et al.*, 2015). Even though perception of mastery focuses on the degree to which a carer is content with the job (Table 1), and the scale used by Sjøgren *et al*. focuses on factors that gives a “troubled conscience,” (Glasberg *et al.*, 2006) these two scales have overlapping themes, and it seems reasonable to argue that to work in a unit where a carer often has a troubled conscience is the opposite to being content with their work.

One can also assume that the perception of mastery is closely linked to job satisfaction, but as job satisfaction remained strongly associated to PCC even when adjusted for all QPS-Nordic subscales, job satisfaction probably includes more than just perception of mastery.

Several studies enhance the role of leaders in promoting PCC. Brownie and Nancarrow found in a review that leadership is important in culture-change processes towards PCC, as introduction of democratized approaches to decision-making that involve residents and staff (Brownie and Nancarrow, 2013). Rokstad *et al*. found that leaders have a central role in drawing up a clear and consistent professional vision, being continuously supportive to the care staff and taking an active part in the care practice as role models when implementing PCC using Dementia Care Mapping (DCM) in nursing homes (Rokstad *et al.*, 2015). The finding in the present study shows that especially “empowering leadership” is associated with PCC. Empowering leadership is a managerial style supporting and encouraging the caregivers to take the initiative and to participate in decisions. Thus, the caregivers closest to the patients have greater influence on making decisions regarding daily care. These findings are in line with the findings from a literature review conducted by Brownie and Nancarrow, who concluded that the introduction of democratized approaches to decision-making involving both staff and patients and models focusing on staff empowerment are important elements to support PCC (Brownie and Nancarrow, 2013). Kitwood also underlined the importance of staff being free to take their own decisions when taking care of persons with dementia and described this as an important part of conducting PCC (Kitwood, 1997).

In the present study, an innovative climate was associated with PCC. To our knowledge, this finding has not been reported earlier. However, the components in an innovative climate, such as taking the initiative and encouraging staff to find alternative ways to do things, corresponds with the theory of PCC (Kitwood, 1997; Brooker, 2004).

Furthermore, the present study showed that the perception of group work was positively associated with PCC. This finding is supported by Hunter *et al.*, who found that collaboration in care is important for PCC (Hunter *et al.*, 2015). Rokstad *et al.* found that leaders who participated in the daily care saw themselves as role models and encouraged the staff more than leaders not taking part in the daily care of the patients (Rokstad *et al.*, 2015). The results of these studies (Hunter *et al.*, 2015; Rokstad *et al.*, 2015) support the finding of a positive association between PCC and empowered staff in the present study.

The positive association between perception of group work and PCC in the present study is supported by Hunter *et al.* (2015). Hunter measured PCC with self-rated measurements, creating five subscales (autonomy, personhood, knowing the person, comfort care, and support for relationships) and found that in four of the five subscales collaboration was the only environmental variable that was associated with PCC. Further, they suggested that focusing on changing organizational processes to create PCC may be more fruitful than a focus on individual behavior, concluding that collaboration in care is important to promote PCC (Hunter *et al.*, 2015).

In a recent literature review, Chaudhury *et al*. (2017) stated that the physical environment of the unit plays an important role in the care of persons with dementia, both in enhancing the patient's quality of life and in the quality of care. In addition, they highlighted the influence of the unit size, the spatial layout, the homelike character, sensory stimulation, and specific spaces on the patients’ behavior and well-being, but they emphasized that the potential of a therapeutic physical milieu is meaningfully utilized only when taking into account the unit's organizational policies and relational care practices (Chaudhury *et al.*, 2017). These findings are supported by Brownie and Nancarrow, who in a review investigated the evidence for the impact of person-centered interventions on aged-care residents and nursing-staff support, concluding that person-centered interventions are multifactorial, where elements of environmental enhancement are included (Brownie and Nancarrow, 2013). The findings in the present study support this conclusion. As the assessment of the physical environment (measured with the SCUEQS) remains significantly associated with PCC after adjusting for size and type of ward, other variables describing the physical environment are as important as size of ward when the staff assess PCC with P-CAT. It is also worth highlighting that “Public areas homelike” was the variable that differs most between SCUs and RUs (Table 2).

It is important to underline that the staff variables and the QPS-N variables contributed more to the model than the environmental variables (Table 3, R^2^_1_and R^2^_2_) and indicated that it is important to focus on these factors independent of type of ward.

### Strengths and weaknesses

To our knowledge, this is the first study exploring the complex associations between PCC, organizational, NH staff and unit characteristics in Norwegian NH. The strengths of this study are the large number of NH units and care staff that participated and the use of standardized and reliable assessment tools which made it possible to compare findings with other studies, both in Norway and internationally. The high number of care staff included in the study is also a strength, enabling inclusion of several potential important variables in the regression analyses. The distribution of the care-staff questionnaire was done in cooperation with the head nurse of the unit, and the procedure for returning the answers enhanced the care staff's anonymity. The physical environmental assessments (TESS-NH) were conducted by five researchers, which have had the same training doing the TESS-HN. The NH staff had no information about the score of the physical environment when scoring the P-CAT. The high response rate from both the leaders of the unit and the care staff, 99.5% and 67.5%, respectively, is a strength of the study.

There are number of limitations to this study, and we consider the complex causal pathways as the most important. The NH staff rated both PCC, their work-related psychosocial factors and job satisfaction, giving only the staffs’ perception and possibly leading to biased data. A limitation of the study is that the participating units were not selected randomly, but they were a convenient sample of NHs geographically distributed throughout Norway and representing small and large units, SCUs and RUs. We used the SCUEQS to assess the physical environment in the NH, which is an American scale developed in 2002. Although there may be differences due to cultural issues between Norway and the U.S., using a standardized instrument gives better data quality than just select arbitrary environmental variables. Thus, the SCUEQS scale is the most proper environmental scale for this study.

## Conclusion

The association between PCC and several organizational, NH staff and unit characteristics identified in this study indicates that providing PCC in NH care is closely linked to how the staff experiences their job situation in addition to both organizational and structural factors and the physical environment. This knowledge is important for creating better care for persons with dementia, and attention needs to be given to this when planning NH care.

## Conflict of interest

None.

## Description of authors’ roles

I. Røen designed the study, collected the data, and wrote the paper. Ø. Kirkevold was involved in designing the study, performed the statistical analysis, and assisted with the writing. I. Testad assisted with the writing and revised the article critically. G. Selbæk was involved in designing the study and revised the article critically for important intellectual content. K. Engedal revised the article critically for important intellectual content. S. Bergh was involved in designing the study, supervised the data collection, and assisted with the writing. All authors approved the final version.
